# Expanding HIV/AIDS care service sites: a cross sectional survey of community pharmacists’ views in South-East, Nigeria

**DOI:** 10.1186/s40545-017-0122-x

**Published:** 2017-11-02

**Authors:** Nnenna Ajagu, Maureen Ugonwa Anetoh, Sunday Odunke Nduka

**Affiliations:** Department of Clinical Pharmacy and Pharmacy Management, Faculty of Pharmaceutical Sciences, Nnamdi Azikiwe University, Awka, Anambra State Nigeria

**Keywords:** Community pharmacists, HIV services sites, Attitude, Willingness and readiness

## Abstract

**Background:**

Community pharmacists are very accessible to most patients yet; they have been underutilized in the aspect of providing HIV care and services. The World Health Organisation recently recommended expanding community pharmacists’ roles to address the increasing complexity of antiretroviral agents and co-infection drug regimen. This study therefore was designed to assess the readiness and willingness of community pharmacists in Nigeria to participate in the care of people living with HIV/AIDS and the possible inclusion of their pharmacy premises as sites for HIV care services.

**Methods:**

A descriptive cross sectional survey was carried out among 205 community pharmacists in south east, Nigeria between October, 2016 and February, 2017. Two hundred and five self-administered questionnaires were distributed to conveniently selected community pharmacists in the region. Data collected were analysed using SPSS version 23. Descriptive statistics was conducted for the demographics and percentage mean scores for each domain were computed. The variables in each domain were categorised into groups and simple percentages were used to show the percentage distribution of the variables. Cross tabulation was also carried out to show the relationship between the variables and groups’ differences were explored using analysis of variance and *P*-values <0.05 were considered significant.

**Results:**

All distributed questionnaires were filled and retrieved. The overall knowledge of HIV among the surveyed pharmacists was seen to be high (70.41%). Although the percentage attitude score of the respondents towards HIV care services was on the average (57%), they were highly willing and ready to use their premise to offer HIV services with a percentage mean readiness score of 87.32%. However, their perceived skills in carrying out these services were observed to be low.

**Conclusion:**

Community pharmacists in the south eastern part of Nigeria have high knowledge of HIV and a somewhat attitude towards HIV care services with high willingness and readiness to be involved in HIV care and services. Despite efforts to engage community pharmacists in HIV services more is needed in the aspect of making adequate policies to further empower more community pharmacists in this aspect of care.

**Electronic supplementary material:**

The online version of this article (10.1186/s40545-017-0122-x) contains supplementary material, which is available to authorized users.

## Background

HIV infection continues to be a major global public health concern having claimed more than 35 million people worldwide [1] mostly in the developing countries and sub-Saharan Africa in particular [2]. Voluntary counseling and testing together with access to antiretroviral agents have been identified as the key strategies in HIV/AIDS prevention and survival of infected individuals [3]. Expanding universal access to these strategies are therefore, the critical steps to achieving HIV/AIDS vision 2020 which aim to end the AIDS epidemic by 2030 [4].

Effective implementation of these critical steps have been hindered by some factors including accessibility of facilities, high cost of transportation to the clinics, long waiting times and lack of flexibility of HIV clinics among others [5]. Of about 36.9 million people living with HIV/AIDS (PLWHA) globally, an estimate of 42.8% had access to antiretroviral therapy in 2014 [6]. Sub-Saharan Africa which account for about 71% of the global estimate of PLWHA had 37% access and Nigeria only 22% with just about 740, 000 out of 3.4 million infected people currently under treatment [6, 7]. Furthermore, only about 54% of people with HIV worldwide know their status with little number recorded in low- and middle-income countries [8]. According to UNAIDS, about 95% of HIV service delivery takes place in the Hospitals [9]. The World Health Organization drawing on the Cochrane Review results recommended initiating HIV care and services at peripheral clinics and in community settings [10] as part of the strategies needed to close the HIV testing and counseling gap especially in sub-Saharan Africa [11]. This strategy involves the decentralization of HIV services from hospitals to more peripheral health facilities [10]. Community pharmacy settings have been recognized as effective sites for health promotion, screening and chronic diseases management [12] and could effectively be utilized in the decentralization strategy. Thus, WHO recommended expanding the roles of community pharmacists to increase access to HIV services [13]. A cross sectional survey of 207 randomly selected community pharmacists in Pune, India between 2004 and 2005 showed a low knowledge and poor attitude towards HIV infected patients but a high willingness to participate in the provision of care to HIV infected persons [14]. Another study in Spain that randomly surveyed the use of HIV rapid screening tests in 20 pharmacies also showed that patients reported that the convenience and accessibility of community pharmacies were significant motivators for their decision to get tested [15]. Recently, the Pharmaceutical Society of Nigeria (PSN) president signed a memorandum of understanding on strengthening integrated delivery services on HIV/AIDS with Howard University Pace Centre (HUPACE) in Nigeria where he highlighted that the community pharmacists will be involved with testing, counseling, care support services and distribution of antiretroviral agents. Although a model for community pharmacists’ involvement in HIV/AIDS services has been proposed in Nigeria [16], there is still need to access the community pharmacists’ readiness and level of interest in these services. This study therefore, was designed to access the readiness and willingness of community pharmacists in South east, Nigeria to participate in HIV care and services. The study further evaluated the pharmacists’ knowledge, attitude and skills with their present level of involvement in these services.

## Methods

### Study population and study design

The study is a cross sectional study carried out among registered community pharmacists in the South east states of Nigeria between October, 2016 and February, 2017. The South east Nigeria is one of the 6 geo-political zones in Nigeria and mainly, the indigenous homeland of the Igbo ethnic group [17]. It is made up of 5 states namely; Anambra, Abia, Imo, Enugu, and Ebonyi states. According to the 2006 population census in Nigeria, this zone has an estimated population of about 16,381,729 people [18]. In addition to the numerous health institutions in the zone including primary, secondary and tertiary health institutions, community pharmacies and community pharmacists forms major part of healthcare facilities and key health care personnel in this zone [19].

According to the information from the various state offices of the Pharmacists Council of Nigeria (PCN) in the region, there were about 441 registered community pharmacists (98 in Anambra state, 103 in Imo state, 125 in Enugu state, 41 in Ebonyi state and 74 in Abia state) in south east Nigeria as at the time of carrying out the study. Using the number of community pharmacists registered in each state and assuming a confidence level of 95% with a confidence interval of +/− 12 in each case, a sample size of 35 for Abia state, 40 for Anambara state, 44 for Enugu state, 26 for Ebonyi state and 41 for Imo state with a total number of 186 were deemed appropriate for the study [20]. An additional 10% of the total calculated sample size (186) distributed in the ratio of 2:3:3:1:2 to Anambra, Imo, Enugu, Ebonyi and Abia states respectively with an extra one respondent added to Enugu state were included to accommodate non-usable questionnaires due to improper filling.

A total of 205 questionnaires were conveniently distributed in the zone as follows: Abia state, 38; Anambra state, 43; Enugu state, 50; Ebonyi state, 28 and Imo state, 46.

The state chairman of Association of Community Pharmacists of Nigeria (ACPN) in each of the five states was contacted to find out their respective dates for 2016 annual general meeting. The annual general meeting day was considered and used because virtually all members of the association will be in attendance. Using the estimated sample size from each state, questionnaires were distributed to selected members in attendance that orally consented until the projected number in the state was reached. Therefore, a total of 205 questionnaires were distributed and retrieved throughout the 5 states. The inclusion criteria are registered community pharmacists who are currently practicing while pharmacists practicing in other sectors and intern pharmacists are excluded from the study.

### Research instrument and validation

A 31-item self-administered questionnaire was developed, validated and used for the study (Additional file 1). The questionnaire was developed by reviewing various documents on HIV/AIDS and previous published works [14, 21–25].

The instrument was structured to consist of four different sections. The first section included socio-demographic characteristics. The second section consisted of seven questions that assessed respondents’ knowledge of HIV and antiretroviral agents (ARVs). Section three contained information used to evaluate the community pharmacists’ attitude and present involvement in HIV care and services. This section consisted of ten questions, with three questions making up the attitude domain and that of involvement constituting seven questions. The last section (section 4) assessed readiness and their perceived skills in HIV services. Four questions assessed readiness while five questions assessed their perceived skills.

The survey instrument was independently face validated by three pharmacy lecturers in the department of Clinical Pharmacy and Pharmacy Management, Nnamdi Azikiwe University, Awka, two Hospital Pharmacists specially trained in the care of HIV-infected patients and one Family Health International (fhi360) staff in Anambra state. The instrument was then, pilot tested to assess its feasibility in Asaba, Delta state. Asaba is the capital of Delta state which is one of the states in the South-south geopolitical zone of Nigeria. It has common boundaries with Anambra state and has a cluster of community pharmacies and a good number of pharmacists. The questionnaire was distributed to 15 community pharmacists after getting their consent. Retrieved questionnaires were coded, entered into SPSS 23 and the internal consistency of the questions was checked using Cronbach’s alpha (CA) with an alpha value of ≥0.70 considered reliable. The corrected item-total correlation of each item was calculated and items with a corrected item-total correlation value of ≥0.3 were retained. The result from the pilot was used to modify the questionnaire to further improve the instrument. During the modification, a total of seven questions were deleted and questions especially in the skills section changed from a ranged question to a “Yes” or “No” response.

### Ethical consideration

Ethical approval was obtained from the ethics committee of Nnamdi Azikiwe University Teaching Hospital, Nnewi, Anambra state, Nigeria with approval number, **NAUTH/CS/VOL.9/132/2016/106**. The study secured oral informed consent from the respondents. Anonymity of participants’ data was maintained by not including participants’ name, name and address of their pharmacies.

### Data analysis

Retrieved copies of the questionnaire were collected and classified into domains. Each domain was coded and entered into excel spread sheet and analysed using SPSS version 23. Descriptive statistics was conducted for the demographics and percentage mean scores for each domain were computed. The variables in each domain were categorised into groups and simple percentages. Cross tabulation was also carried out to show the relationship between the variables. The groups’ differences were explored using analysis of variance (ANOVA) at *P* < 0.05.

The knowledge domain consisted of 15 expanded items with ‘yes’ or ‘no’ responses, some of which were negatively worded. Each correctly answered item was scored ‘1’ and ‘0’ if otherwise, giving possible minimum and maximum sum total scores of 0 and 15 for knowledge. The total correct answers were transformed into percentage knowledge scores. For ease of comparison, the knowledge status were divided into ‘low’, ‘moderate’ and ‘high’ knowledge based on their percentage scores. Respondents who scored below 50 were categorized as having low knowledge, respondents who scored between 50.00 and 69.99 were categorized as having moderate knowledge while respondents who had equal or greater than 70 were categorized as having high knowledge.

The involvement domain consisted of 9 expanded items with ‘Yes’ or ‘No’ responses, where a ‘Yes’ response indicated ‘involved’ and a ‘No’ indicated ‘not involved’. Each item activity where the respondent was involved was scored ‘1’ and ‘0’ if otherwise, giving possible minimum and maximum sum total scores of 0 and 9 involvement domain respectively. The total score of involvement was transformed into percentage involvement scores. For ease of comparison, the involvement status were further divided into ‘low’, ‘moderate’ and ‘high’ involvement based on their percentage scores. Respondents who scored below 50 were categorized as having low involvement, respondents who scored between 50.00 and 69.99 were categorized as having moderate involvement while respondents who had equal or greater than 70 were categorized as having high involvement.

The attitude domain comprised 5 items with 5-likert scale of ‘strongly disagree, disagree, undecided, agree and strongly agree’. The strongly disagree was scored ‘0’, disagree ‘1’, undecided ‘2’, agree ‘3’ and strongly agree ‘4’. The items were worded to reflect ‘negative’ to ‘positive’ attitude when responses from ‘strongly disagree’ to ‘strongly agree’ were selected. This gave possible minimum and maximum sum total scores of 0 and 20 for attitude respectively. These total scores were transformed into percentage attitude scores. For ease of comparison, the attitude status was divided into ‘negative’ and ‘positive’ based on the percentage mean attitude scores. Respondents who scored below the mean were categorized as having negative attitude while respondents who had equal or greater than the mean were categorized as having positive attitude.

The readiness domain consisted of 4 items with ‘Yes’ response indicating ‘ready’ and a ‘No’ response indicating ‘not ready’. Readiness was scored ‘1’ and ‘0’ if otherwise, given possible minimum and maximum sum total scores of 0 and 4 for readiness. The total scores were transformed into percentage readiness scores. For ease of comparison, the readiness status were divided into ‘low’, ‘moderate’ and ‘high’ readiness based on their percentage scores, respondents who scored below 50 were categorized as having low readiness, respondents who scored between 50.00 and 69.99 were categorized as having moderately ready while respondents who had equal or greater than 70 were categorized as being highly ready.

The perceived skill domain consisted of 5 items with ‘Skilled’ or ‘not skilled’ responses. Skilled response was scored ‘1’ and ‘0’ if otherwise, given possible minimum and maximum sum total scores of 0 and 5 for skill. The total scores were transformed into percentage skill scores. For ease of comparison, the skill status were divided into ‘low’, ‘moderate’ and ‘high’ knowledge based on their percentage scores, respondents who scored below 50 were categorized as having low skill, respondents who scored between 50.00 and 69.99 were categorized as having moderate skill while respondents who had equal or greater than 70 were categorized as being highly skilled.

## Result

The demographic characteristics of the respondents (Table 1) showed that majority of the community pharmacists were male (61%) with more than half of them within the age range of 31-50 years. While most of the pharmacists (52.7%) have ≤10 years of experience, a good number of them (63.4%) have about 5 or less than five employees in their premises.

**Table 1:** Demographic characteristics of respondents

Variable	Frequency (%)
Gender	
Male	125 (61.0)
Female	80 (39.0)
Age	
≤ 30	23 (11.2)
31–40	80 (39.0)
41–50	46 (22.4)
51–60	38 (18.5)
≥ 60	18 (18.8)
Number of employees	
≤ 5	130 (63.4)
6–10	60 (29.3)
11–15	7 (3.4)
> 16	8 (3.9)
Years of practice	
< 5	49 (23.9)
5–10	59 (28.8)
11–15	31 (15.5)
16–20	14 (6.8)
> 20	52 (25.4)
Qualifications	
B. Pharm	128 (62.4)
M. Pharm	48 (23.4)
Pharm.D	23 (11.2)
FPCPharm	3 (1.5)
MBA	3 (1.5)

Item analysis of the community pharmacists’ responses to HIV and HIV services are shown in Table 2. The pharmacists showed a very good knowledge of HIV with a percentage mean score of 70.41%. While majority of the respondents were able to get most questions to HIV knowledge, 70.2% did not seem to understand the WHO clinical staging of this disease. A large number of the respondents (96.6%) showed a positive attitude towards community pharmacists’ role(s) in the provision of HIV services with about two-third (62.9%) showing willingness for the use of their premises for the provision of these services. However, the percentage mean attitude score of the respondents was 57.0%**.** With a mean involvement score of 36.72%, the surveyed community pharmacists’ were seen not to be currently involved in HIV services as more than two-third revealed that they do not encounter HIV clients in their premises. Although most community pharmacists stock condoms (90.7%), just very few of them stock HIV test kits (23.4%) and antiretroviral agents (31.7%). Assessment of community pharmacists readiness to offer HIV services gave a mean response score of 87.32% with majority (>90%) having backup generator and counselling section/room. The community pharmacists perceived skill score in carrying out HIV/AIDS services was poor with a percentage mean score of 39.26%.

**Table 2:** Respondents assessment

Variable	Response (%)	
Knowledge	Wrong	Correct
Goal(s) for HIV therapy		
Prolongation of quality of life	23 (11.2)	182 (88.8)
Achievement of immune reconstitution	74 (36.1)	131 (63.9)
Reduction in HIV transmission	41 (20.0)	164 (80.0)
Reduction in viral load	35 (17.1)	170 (82.9)
Criteria for initiating HAART		
CD4 level 500 or below	46 (23.9)	159 (76.1)
WHO Stage 3 and 4 irrespective of CD4 count	79 (38.9)	126 (61.5)
Co-existing TB Infection	86 (42.0)	119 (58.0)
Understanding of the WHO clinical staging of HIV/AID	144 (70.2)	61 (29.8)
Minimum number of ARVs that should be included in an ideal regimen	51 (24.9)	154 (75.1)
Approved combination of ARVs used in HAART regimen		
Efavirenz + Lamivudine + Tenofovir	73 (35.6)	132 (64.4)
Abacavir + Lamivudine + Efavirenz	112 (54.6)	93 (45.4)
Lopinavir/ritonavir + Lamuvidine +Zidovudine	82 (40.0)	123 (60)
Zidovudine + Lamuvudine + Emtricitabine	27 (13.2)	178 (86.8)
Drug added as booster to Lopinavir	95 (46.3)	110 (53.7)
Ideal storage condition for ARV	54 (26.3)	151 (73.7)
*Percentage mean knowledge score = 70.41 ± SD (19.09)*		
Involvement	Involved	Not Involved
HIV clients encounter in your premise	48 (23.4)	156 (76.1)
Activities with HIV clients in your premise	47 (22.9)	157 (76.6)
Activities with suspected HIV clients in your premise	34 (16.6)	170 (82.9)
Educate, counsel and reassure the patient	59 (28.8)	144 (70.2)
Manage the patient for other associated minor conditions and document	8 (3.9)	195 (95.1)
Refer patient to hospital	143 (69.8)	60 (29.3)
Stock HIV test kit	48 (23.4)	157 (76.6)
Stock ARVs	65 (31.7)	140 (68.3)
Stock Condoms	186 (90.7)	19 (9.3)
*Percentage mean involvement score = 36.72 ± SD (17.34)*		
Attitude	Positive attitude	Negative attitude
Community pharmacist have a role to play in provision of HIV/AIDS Services	198 (96.6)	7 (3.4)
Wiilingness to use your premise for the provision of HIV services	129 (62.9)	75 (36.6)
Factor that will enhance willingness to offer HIV services		
Training and sensitization lectures are necessary to enhance community pharmacists involvement in HIV/AIDS care	182 (88.8)	22 (10.7)
Payment/remuneration	104 (50.7)	100 (48.8)
other incentives	62 (30.2)	142 (69.3)
*Percentage mean Attitude score = 56.98 ± SD (19.67)*		
Readiness	Ready	Not ready
Functional backup generator	194 (94.6)	11 (5.4)
Counseling section	199 (97.1)	6 (2.9)
Counseling section with auditory and visual privacy	155 (77.5)	50 (22.5)
Use of Patient information sheet/book	136 (66.6)	69 (33.7)
*Percentage mean readiness score = 87.32 ± SD (15.34)*		
Perceived Skill	Not Skilled	Skilled
Educating target population on HIV	112 (54.6)	92 (44.9)
Demonstrable skill on how to carry out HIV rapid test	150 (73.2)	54 (26.3)
Counselling on importance of adherence to HIV medication	117 (57.1)	87 (42.4)
Referral of HIV client for care	123 (60.0)	81 (39.5)
HIV care and documentation and reporting	144 (70.2)	61 (29.8)
*Percentage mean skill score = 39.26 ± SD (38.13)*		

The impact of demographic characteristics on HIV knowledge of the respondents is shown in Table 3. A one way between groups analysis of variance with knowledge divided into low, middle and high knowledge levels showed a statistical significant difference at the *p* < 0.001 for the demographic characteristics F(4, 200) = 5.23, *p* < 0.001 with a moderate effect size (0.09). Independent t-test analysis indicated that the mean knowledge score for the female gender (M = 70.13 ± SD(70.93) was not significantly different from that of the male (M = 68.67 ± SD(17.67). On the age distribution, post hoc analysis using Dennett’s test indicated that the mean knowledge score for >60 years age group (M = 58.33 ± SD(24.27) was significantly different from those of 31–40 years (M = 75.69 ± SD(17.89, *p* < 0.001) and 45–50 years (M = 73.19 ± SD(17.06), *p* = 0.01) but not significantly different from the others. Similarly, comparison of knowledge level and respondents years of practice using 20 yrs. of practice as referent indicated a significant mean knowledge difference between the referent group and those of 6–10 years in practice (*p* < 0.001).

**Table 3:** Knowledge of respondents based on demographic characteristics

Demographic characteristics	Low knowledge n (%)	Moderate knowledge n (%)	High knowledge n (%)	Mean	Std. deviation	Std. error	*P* value
Gender							
Male	23 (65.7)	38 (30.4)	64 (56.6)	68.67	17.67	1.58	0.12^a^
Female	12 (34.3)	19 (33.3)	49 (43.4)	73.13	20.93	2.34	
Age (Years)							
≤ 30	5 (14.3)	6 (10.5)	12 (10.6)	64.98	23.21	4.81	0.53^b^
31–40	10 (28.6)	16 (28.1)	54 (47.8)	75.69	17.89	2.00	<0.001^b^
41–50	5 (14.3)	14 (24.6)	27 (23.9)	73.19	17.06	2.52	0.01^b^
51–60	6 (17.1)	19 (33.3)	13 (11.5)	64.91	13.98	2.27	0.46^b^
≥ 61	9 (25.7)	2 (3.5)	7 (6.2)	58.33	24.27	5.72	Referent
Number of employees							
≤ 5 staff	30 (85.7)	36 (63.2)	64 (56.6)	66.45	19.21	1.68	0.77^b^
6–10 staff	4 (11.4)	18 (31.6)	38 (33.6)	77.59	16.27	2.10	0.55^b^
11–15 staff	0 (0.0)	1 (1.8)	6 (5.3)	81.75	12.30	4.65	0.44^b^
≥ 15 staff	1 (2.9)	2 (3.5)	5 (4.4)	70.83	24.44	8.64	Referent
Years of Practice (Years)							
≤ 5	12 (34.3)	13 (22.8)	24 (21.2)	64.83	23.44	3.35	0.99^b^
6–10	3 (8.6)	12 (21.1)	44 (38.9)	78.63	14.55	1.89	<0.001^b^
11–15	5 (14.3)	10 (17.5)	16 (14.2)	17.15	17.00	3.05	0.28^b^
16–19	0 (0.0)	4 (7.0)	10 (8.8)	76.98	10.42	2.78	0.07^b^
≥ 20	15 (42.9)	18 (31.6)	19 (16.8)	64.10	18.49	2.56	Referent
Qualification(s)							
B. Pharm	32 (91.4)	40 (70.2)	56 (49.6)	64.83	18.59	1.64	0.27^b^
M. Pharm	3 (8.6)	15 (26.6)	30 (26.5)	73.94	16.21	2.34	0.85^b^
Pharm. D	0 (0.0)	1 (1.8)	22 (19.5)	90.33	9.95	2.07	0.54^b^
FPC. Pharm	0 (0.0)	0 (0.0)	3 (2.7)	88.89	5.56	3.21	0.78^b^
MBA	0 (0.0)	1 (1.8)	2 (1.8)	79.62	21.03	12.14	Referent

^a^t-test was applied, ^b^Dunnet test was applied, *n* = 205

The impact of demographic characteristics on the respondents’ level of involvement in HIV/AIDS activities in their community pharmacies are presented in Table 4 with the involvement level grouped into low, moderate and high involvement with a statistical significant difference seen at *p* = 0.039 for the demographic characteristics F (4, 197) = 2.57, *p* < 0.039. However, the actual difference in mean scores between groups calculated from the eta squared was quite small (0.04). Whereas no significant difference was seen between the mean involvement scores of the male and female gender (*p* > 0.05), significant difference was seen between the mean involvement score for the referent age group (M = 25.25 ± SD(16.95) and the other age groups. Post hoc analysis indicated a significant difference between the involvement mean score for those with MBA (M = 69.69, ±SD(21.00) and those with B.Pharm (M = 35.85 ± SD(16.76), *p* < 0.001), M.Pharm (M = 41.67 ± SD(17.27), *p* = 0.01) and Pharm D (M = 26.48 ± SD(12.53), *p* < 0.001).

**Table 4:** Involvement of respondents based on demographic characteristics

Demographic Characteristics	Low involvement n (%)	Moderate involvement n (%)	High involvement n (%)	Mean	Std. deviation	Std. error	*p* value
Gender							
Male	93 (59.6)	25 (69.4)	4 (40.0)	38.15	15.58	1.41	0.15^a^
Female	63 (40.4)	11 (30.6)	6 (60.0)	34.55	19.62	2.20	
Age (Years)							
≤ 30	17 (10.9)	5 (10.9)	1 (10.0)	41.50	*13.93*	*2.90*	0.01^b^
31–40	54 (36.4)	21 (58.3)	5 (50.0)	37.85	*19.70*	*2.20*	0.02^b^
41–50	35 (22.4)	9 (25.0)	1 (10.0)	36.57	15.56	2.30	0.05^b^
51–60	33 (21.2)	1 (2.8)	2 (20.0)	37.12	*14.15*	*2.35*	*0.05* ^*b*^
≥ 61	17 (10.9)	0 (0.0)	1 (10.0)	25.25	16.95	4.00	Referent
No of employees							
≤ 5 staff	97 (62.2)	27 (75.0)	3 (30.0)	35.93	16.47	1.46	0.34^b^
6–10 staff	47 (30.1)	8 (22.2)	5 (50.0)	36.95	19.15	2.47	0.45^b^
11–15 staff	6 (3.8)	1 (2.8)	0 (0.0)	40.26	11.57	4.37	0.89^b^
≥ 15 staff	6 (3.8)	0 (0.0)	2 (20.0)	44.32	21.42	7.57	Referent
Years of practice (Years)							
≤ 5	40 (25.6)	8 (22.2)	1 (10.0)	36.18	16.75	2.39	0.99^b^
6–10	42 (26.9)	13 (36.1)	4 (40.0)	36.36	18.87	2.46	0.99^b^
11–15	22 (14.1)	8 (22.2)	0 (0.0)	38.18	14.99	2.74	0.85^b^
16–19	9 (5.8)	4 (11.1)	1 (10.0)	42.21	16.59	4.43	0.52^b^
≥ 20	43 (27.6)	3 (8.3)	4 (40.0)	35.27	17.87	2.53	Referent
Qualifcation(s)							
B. Pharm	98 (62.8)	22 (61.1)	5 (50.0)	35.85	16.78	1.5	<0.001^b^
M. Pharm	32 (66.7)	13 (36.1)	3 (30.0)	41.67	17.27	2.39	0.01^b^
Pharm. D	22 (14.1)	1 (2.8)	0 (0.0)	26.48	12.53	2.61	<0.001^b^
FPC.Pharm	3 (1.9)	0 (0.0)	0 (0.0)	39.39	10.50	6.06	0.06^b^
MBA	1 (0.6)	0 (0.0)	2 (20.0)	69.69	21 .00	12.12	Referent

^a^t-test was applied, ^b^Dunnet test was applied, *n* = 205

On the assessment of respondents attitudes based on demographic characteristics (Table 5), more male than female showed positive attitude towards HIV/AIDS care and services in the community pharmacies with respective mean scores of 58.94 ± SD(20.05) and 53.95 ± SD(18.79). However, their difference was not significant (*p* > 0.05). About 25% of the respondents with less than 5 years of practice showed negative attitude towards HIV/AIDS, with 63% with B.Pharm qualification showing a positive attitude towards HIV services. A correlation between respondents’ attitude and their readiness towards HIV services was seen to be statistically significant (*p* < 0.05).

**Table 5:** Attitude of respondents based on demographic characteristics

Demographic characteristics	Negative Attitude n (%)	Positive attitude n (%)	Mean	Std. deviation	Std. error	*p* value
Gender						
Male	67 (56.3)	56 (66.7)	58.94	20.05	1.81	0.08^a^
Female	52 (43.7)	28 (33.3)	53.95	18.79	2.11	
Age (Years)						
≤ 30	13 (10.9)	10 (11.9)	55.07	22.72	4.74	0.55^b^
31–40	42 (35.3)	38 (42.2)	57.71	17.58	1.97	0.16^b^
41–50	26 (21.8)	19 (22.6)	59.26	19.96	2.98	0.12^b^
51–60	23 (19.3)	14 (16.7)	58.11	21.38	3.52	0.19^b^
≥ 61	15 (12.6)	3 (3.6)	48.15	19.71	4.65	Referent
No of employees						
≤ 5 staff	89 (74.8)	39 (46.4)	52.73	18.21	1.61	0.09^b^
6–10 staff	25 (21.0)	35 (41.7)	64.44	19.03	2.46	0.96^b^
11–15 staff	4 (3.4)	3 (3.6)	59.52	26.97	10.19	0.72^b^
≥ 15 staff	1 (0.8)	7 (8.3)	66.67	23.57	8.33	Referent
Years of Practice (Years)						
≤ 5	34 (26.8)	15 (17.9)	54.08	16.85	2.41	0.66^b^
6–10	27 (22.7)	32 (38.1)	59.04	19.41	2.53	1.00^b^
11–15	18 (15.1)	12 (14.3)	56.11	20.75	3.79	0.97^b^
16–19	8 (6.7)	6 (7.1)	54.76	23.05	6.16	0.94^b^
≥ 20	32 (26.9)	19 (22.6)	58.50	21.80	2.97	Referent
Qualification(s)						
B. Pharm	75 (63.0)	51 (60.7)	55.69	19.80	1.76	0.29^b^
M. Pharm	30 (21.2)	18 (21.4)	5.66	20.26	2.93	0.34^b^
Pharm. D	12 (10.1)	11 (13.1)	61.59	15.44	3.22	0.63^b^
FPC.Pharm	1 (0.8)	2 (2.4)	66.67	28.87	16.67	0.96^b^
MBA	1 (0.8)	1 (0.8)	72.22	25.46	14.70	Referent

^a^t-test was applied, ^b^Dunnet test was applied, *n* = 205

Impact of demographic characteristics on the respondents’ level of readiness in HIV/AIDS activities in their community pharmacies are presented in Table 6 with the readiness level grouped into low, moderate and high readiness. Both gender groups were highly ready to offer HIV services in their premise with a mean score of 86.8 ± SD (15.48) for female and 87.66 ± SD (15.96) for male. Encouragingly, majority of those with B.Pharm qualification were highly ready to offer HIV services 85.26 ± SD(11.39). Table 7 shows the impact of demographic characteristics on the respondents’ skill in HIV/AIDS activities in their community pharmacies with skill grouped into low, moderate and high skilled with an effect size of 0.07 for the demographic characteristics F (4, 199) = 3.75, *p* < 0.001. On the age distribution, post hoc analysis using Dunnett’s test indicated that the mean knowledge score for >61 years age group (M = 25.56 ± SD(34.85) was significantly different from those of 51–60 years (M = 51.31 ± (SD)32.98, *p* = 0.04). Also, the distribution of number of employee using post hoc analysis revealed that the mean skill score for employee ≥15 (M = 72.50 ± SD(44.96) was significantly different from those ≤5 (M = 38.15 ± SD(38.71), *p* = 0.03) and those of 6–10 (M = 36.10 ± SD(35.67), p = 0.03). A correlation between the respondents’ skills and their involvement in HIV services was seen to be statistically significant (*p* < 0.05).

**Table 6:** Readiness of respondents based on demographic characteristics

Demographic characteristics	Low readiness n (%)	Moderately ready n (%)	Highly ready n (%)	Mean	Std. deviation	Std. error	*p* value
Gender							
Male	2 (66.7)	13 (61.9)	110 (61.1)	87.66	15.96	1.42	0.71^a^
Female	1 (33.3)	8 (38.1)	70 (38.9)	86.80	15.48	1.74	
Age (Years)							
≤ 3	0 (0.0)	2 (9.5)	21 (11.7)	91.93	14.17	2.96	0.36^b^
31–40	1 (33.3)	6 (28.6)	73 (40.6)	87.14	15.01	1.68	0.92^b^
41–50	2 (66.7)	2 (9.5)	41 (22.8)	89.21	16.45	2.45	0.99^b^
51–60	0 (0.0)	8 (38.1)	30 (16.7)	83.83	16.66	2.70	0.99^b^
≥ 61	0 (0.0)	3 (14.3)	15 (8.3)	84.92	16.60	3.91	Referent
No of employee							
≤ 5 staff	2 (100)	21 (100)	105 (58.3)	84.83	17.38	1.53	0.68^b^
6–10 staff	0 (0.0)	0 (0.0)	60 (33.3)	91.67	10.94	1.41	0.92^b^
11–15 staff	0 (0.0)	0 (0.0)	7 (3.9)	93.88	11.24	4.25	0.83^b^
≥ 15 staff	0 (0.0)	0 (0.0)	8 (4.4)	89.29	14.79	5.23	Referent
Years of Practice (Years)							
≤ 5	1 (33.3)	6 (26.6)	42 (23.3)	86.88	16.71	2.39	0.96^b^
6–10	0 (0.0)	1 (4.8)	57 (31.7)	92.12	11.41	1.50	0.07^b^
11–15	2 (66.7)	6 (28.6)	23 (12.8)	80.18	19.41	3.49	0.44^b^
16–19	0 (0.0)	0 (0.0)	14 (7.8)	92.86	10.85	2.90	0.29^b^
≥ 20	0 (0.0)	8 (21.0)	44 (24.4)	85.16	16.00	2.21	Referent
Qualification(s)							
B. Pharm	2 (66.7)	17 (80.1)	108 (60)	85.26	16.39	1.45	0.84^b^
M. Pharm	1 (33.3)	1 (4.8)	46 (25.6)	90.18	13.87	2.00	1.00^b^
Pharm. D	0 (0.0)	3 (14.3)	20 (11.1)	91.30	15.97	3.33	1.00^b^
FPC. Pharm	0 (0.0)	0 (0.0)	3 (1.7)	95.24	8.25	4.76	0.95^b^
MBA	0 (0.0)	0 (0.0)	3 (1.7)	90.48	8.25	4.76	Referent

^a^t-test was applied, ^b^Dunnet test was applied, *n* = 205

**Table 7:** Skill of respondents based on demographic characteristics

Demographic characteristics	Low skill n (%)	Moderately skillful n (%)	Highly skilled n (%)	Mean	Std. deviation	Std. error	*p* value
Gender							
Male	79 (59.4)	8 (80.0)	37 (60.7)	41.45	36.66	3.29	0.31^a^
Female	54 (40.6)	2 (20.0)	24 (39.3)	35.88	40.31	4.51	
Age (Years)							
≤ 30	12 (9.0)	3 (30.0)	8 (13.1%)	49.13	41.56	8.66	0.12^b^
31–40	61 (45.9)	1 (10.0)	18 (29.5%)	29.73	35.36	3.99	0.97^b^
41–50	26 (19.5)	1 (10.0)	18 (29.5%)	46.44	41.35	6.16	0.12^b^
51–60	20 (15.0)	5 (50.0)	13 (21.3%)	51.31	32.98	5.35	0.04^b^
≥ 61	14 (10.5)	0 (0.0)	4 (6.6%)	25.56	34.85	8.21	Referent
Number of employees							
≤ 5 staff	86 (64.7)	4 (40.0)	40 (65.6)	38.15	*38.71*	*3.35*	0.03^b^
6–10 staff	41 (30.8)	5 (50.0)	13 (21.3)	36.10	*35.67*	*4.64*	0.03^b^
11–15 staff	4 (3.0)	0 (0.0)	3 (4.9)	48.57	38.91	14.71	0.39^b^
≥ 15 staff	2 (1.5)	1 (10.0)	5 (8.2)	72.50	44.96	15.90	Referent
Years of Practice (Years**)**							
≤ 5	33 (24.8)	3 (30.0)	12 (19.7)	33.54	38.4	5.54	0.36^b^
6–10	40 (30.1)	0 (0.0)	19 (31.1)	38.98	38.63	5.03	0.82^b^
11–15	28 (21.1)	0 (0.0)	3 (4.9)	26.77	29.14	5.23	0.11^b^
16–19	3 (2.3)	3 (30.0)	8 (13.1)	65.71	35.24	9.42	0.22^b^
≥ 20	29 (21.8)	4 (40.0)	19 (31.4)	45.91	39.53	5.48	Referent
Qualification(s)							
B. Pharm	84 (63.2)	5 (50.0)	38 (62.3)	38.43	37.97	3.37	0.47^b^
M.Pharm	29 (21.8)	5 (50.0)	14 (23.0)	42.50	38.67	5.58	0.60^b^
Pharm. D	18 (13.5)	0 (0.0)	5 (8.2)	31.17	39.04	8.14	0.33^b^
FPC. Pharm	1 (0.8)	0 (0.0)	2 (3.3)	56.67	49.33	28.48	0.99^b^
MBA	1 (0.8)	0 (0.0)	2 (3.3)	63.33	11.55	6.67	Referent

^a^t-test was applied, ^b^Dunnet test was applied, *n* = 205

## Discussion

This study assessed community pharmacists’ knowledge and attitude together with their present level of involvement in HIV/AIDS care and services including their willingness and readiness to participate in the care and services. HIV/AIDS knowledge among the surveyed community pharmacists was perceived to be high with a somewhat attitude towards the involvement of the pharmacists in HIV/AIDS care and services. Although they are currently not fully involved in this care, they were assessed to be ready to take up these services however; their perceived skills in carrying out these services were poor.

Community pharmacists as the most accessible healthcare professional to the public have been adjudged to be in a better position for early detection and management of chronic diseases together with the other healthcare professionals [26]. Chronic disease screenings, counselling on the appropriate use of medicines, life style changes advocacy, working with other healthcare professionals and general provision of pharmaceutical care services to these patients are some of the identified areas where community pharmacists could help improve the quality of life of patients with chronic diseases [5]. HIV/AIDS is a chronic disease of concern because of its high morbidity and mortality rate. Early detection of HIV and timely initiation of antiretroviral therapy is crucial in achieving the vision of controlling the virus which will help prevent transmission and HIV/AIDS associated deaths [6]. Achieving this is primarily hinged on ensuring universal access to the lifesaving medications and access to other HIV prevention services [4] including counselling and testing. Hence, the use of more peripheral health professional settings including the community pharmacists could be optimally utilized to improve and enhance access to HIV care and services. Although the Global HIV/AIDS initiative Nigeria (GHAIN), launched a programme in 2007 led by Howard University Pharmacist and Continuing Education centre (HU-PACE) through the Pharmacists Council of Nigeria’s (PCN) pharmacy continuing education on skill enhancement among others, the involvement of community pharmacists in HIV/AIDS care and services are still uncertain.

The result of this study showed a high knowledge of HIV by the community pharmacists similar to a study in Mexico [27] but, contrary to studies in India [14] and New York [28] that found low HIV knowledge by community pharmacists. This high knowledge may be attributed to the basic knowledge gained in the universities and through other trainings like the mandatory continuing professional development (MCPD) as well as easy access to information on the internet. Michael and Kristin observed in their study that internet had a positive impact on knowledge level of pharmacists [29]. The incorporation of modules of HIV/AIDS into the Pharmacists Council of Nigeria (PCN) coordinated mandatory continuing professional development (MCPD) for pharmacists in Nigeria may have contributed to the observed high knowledge level. The inability of most of the pharmacists to correctly answer the question on WHO clinical staging of HIV/AIDS was surprising since Nigeria adopted the WHO staging and this has been the basis for the initiation of ART in adults and paediatrics in Nigeria. Further analysis of the pharmacists’ knowledge using post hoc showed that the younger community pharmacists had a significant higher scores than the older ones which still points to the importance of internet in their knowledge base. Moreover, the incorporation of more client based studies in the current curriculum especially with the introduction of pharmaceutical care may have contributed to this observed difference [30]. More so, HIV/AIDS may not have attracted much attention when the present older pharmacists were in school and hence, a possible reduction in their interest on the topic which may have translated to their poorer knowledge. This was further buttressed by the lower knowledge seen among people that have been in practice for a longer time compared to those newer in practice.

Majority of the pharmacists believed that they have major roles to play in the provision of HIV services although they are currently not involved. This may be in line with the FIP statement which recognized pharmacists as stakeholders in the management of chronic diseases as these diseases including HIV/AIDS often require long time use of medicines [26]. Since community pharmacists have been involved in providing pharmaceutical care and in identifying and resolving drug therapy problems including counseling in other chronic diseases, they may have believed that HIV/AIDS should not be an exception hence, the positive outlook. Although most of the community pharmacists are willing to use their premise for these services, they are currently seen not to be involved in the provision of the services with an involvement score of 36.7%. The Global HIV/AIDS imitative Nigeria (GHAIN) in 2007 lunched a program led by Howard University Pharmacist and Continuing Education center (HU-PACE) in some states in Nigeria centered on getting the community pharmacists involved in the provision of care to HIV/AIDS patients but, the program could be adjudged not to be a success. This may be due to the program design and execution mode as the service delivery in the said project was mainly through pharmacist volunteer scheme [31]. Most HIV care services in Nigeria is offered freely through government owned hospitals and for community pharmacists who are in the private sector, without adequate motivation with respect to reimbursement and incentives, their attitude towards providing such services may dwindle because money is needed to maintain the facilities and possibly employ more pharmacists. Since remunerations for the pharmacists were not captured in the pilot program, this may have affected their acceptance of the program and hence, their present low level of involvement seen in this study. This study further found that most of the community pharmacists indicted that reimbursement and incentives will motivate them to offer HIV services in their respective premises. Although the study found some involvement level, this may be just the ones carried out to keep their customers. Moreover, the major area where they were seen to be much involved was in the stocking of condoms which may be for the purpose of family planning. Similar to the findings with the impact of demography on knowledge, the younger pharmacists showed higher level of involvement with HIV services than the older pharmacists which correlates with their knowledge base. This is similar to what was observed in a report on HIV/AIDS services delivery through pharmacist volunteer scheme carried out in Nigeria by Onuoha et al.*,* [31]. Surprisingly, community pharmacists with master’s in business administration showed higher involvement than those with B. Pharm and even Pharm. D. However, the findings of Daly et al., [32] in their study in the University of Buffalo, New York may explain this observation.

Some of the basic requirements by WHO for a facility to be involved in offering HIV care were adequate space, a counselling section with auditory and visual privacy to maintain privacy and confidentiality, as well as a standby generator [33]. These coincidentally form part of the basic requirements for the registration of community pharmacy premises in Nigeria, and as such a boost for the readiness of community pharmacists to offer HIV services [34]. Majority of the community pharmacies used in this study met with these criteria, signifying their readiness to take up such services. Furthermore, about two-third of the pharmacist were willing to use their premise for the provision of HIV services. This is similar to a study carried out in India which showed that community pharmacists in India were highly willing to participate in the care of HIV infected persons [14]. This high willingness to participate in HIV care was also seen in a recent survey conducted by SIDHAS to scale up community based ARV refill in Nigeria [9]. Irrespective of the community pharmacists’ high willingness and readiness to care for the HIV/AIDS patients, they still showed poor skills. This calls for urgent need for further training if they must be involved. Meanwhile, most of the surveyed pharmacists probably recognised this and recommended that training will further enhance their skills and willingness to participate in HIV care and services.

There are a number of limitations in this study. The convenient use of only community pharmacists that attended meetings for the survey may not be a true representative of the population and may not have allowed for an even distribution of the pharmacists according to their practice area. Secondly, the sample size used and the use of only one geo-political zone in Nigeria for the study may not allow for a generalization of the community pharmacists’ views to the entire country. The pharmacists may be bias in assessing their skills since they were asked to rate themselves. Furthermore, the number of items used to assess readiness may not be sufficient to measure pharmacists’ readiness to offer HIV services. Despite these limitations however, this research could serve as a pilot to a more detailed and a larger national survey that will improve community pharmacists’ involvement and participation in the care of people living with HIV/AIDS which will help optimize their roles as well as improve the health of these patients.

## Conclusion

This study observed high knowledge but, low perceived skills of community pharmacists in the study area to care for HIV/AIDS patients. Although they are currently not involved in these services, their high willingness and readiness to get involved is a pointer that training and retraining programs will get them to a better feat of providing these services. Even though a few of them already offer HIV care services, more is needed in the aspects of refill of ARVs, medication treatment and monitoring of ARVs, adherence counseling, social responsibility to HIV clients, stocking of ARVs and HIV test kits and provision of Post Exposure Prophylaxis (PEP). This will help combat some of the hindrances to adherence on HIV care seen in some studies such as lack of accessible HIV care and not having adequate funds to transport to HIV care facilities. We therefore recommend that HIV/AIDS policy makers in Nigeria should expand the coverage of HIV/AIDS services by involving and training the community pharmacists to improve their skills in offering these services.

## Additional file

Additional file 1: Study Instrument. (DOCX 20 kb)

## Abbreviations

GHAIN: The Global HIV/AIDS initiative Nigeria
HU-PACE: Howard University Pharmacist and Continuing Education center
MCPD: Mandatory continuing professional development
PCN: Pharmacists Council of Nigeria
PEP: Post exposure prophylaxis
SIDHAS: Strengthening Integrated Delivery of HIV/AIDS services
