# Supplementary material for: Expanding HIV/AIDS care service sites: a cross sectional survey of community pharmacists’ views in South-East, Nigeria
Source: J Pharm Policy Pract. 2017 Nov 2;10:34. doi: 10.1186/s40545-017-0122-x (PMC5667033; doi:10.1186/s40545-017-0122-x)
Supplement: Additional file 1: — Study Instrument. (DOCX 20 kb) [file 40545_2017_122_MOESM1_ESM.docx]

*Additional file 1: Study Instrument*

**Assessment of Community Pharmacists Willingness and Readiness to Participate in HIV/AIDS**

**Care Services**

There has been increasing efforts to expand HIV/AIDS care service sites to community pharmacies in Nigeria in line with increasing universal access to HIV counseling and testing and ART. This questionnaire is therefore designed to evaluate community pharmacists’ readiness and willingness to participate in this care services in South East, Nigeria. All information supplied here is purely for research purposes and will be kept confidential.

**Please be informed that your participation in this survey is voluntary**.

**Section 1**: **Pharmacist demographics**.

1. **Gender**: Male [ ] Female [ ]
2. **Age** (Years): ≤30 [ ] 31 – 40 [ ] 41 – 50 [ ] 51 – 59 [ ] ≥60 [ ]
3. **Number of employees**: [ ] ≤ 5 [ ] 6 – 10 [ ] 11 – 15 [ ] 16 and above
4. **Years** **in** **practice**: ≤5[ ] 6 – 10 [ ] 11 – 15 [ ] 16 – 19 [ ] ≥20 [ ]
5. **Qualification** (tick all that apply): B.Pharm [ ] M. Pharm [ ] Pharm D [ ] FPC Pharm [ ] MBA [ ]

MPH [ ] PHD [ ]

**Section 2:** **HIV/AIDS Knowledge Assessment**

| **Indicate Yes or No in the options following each question or in the statement(s);** | **Yes** | **No** |
| --- | --- | --- |
| 1. **What is/are the goal(s) for HIV therapy?** |  |  |
| Prolongation of quality of life | [ ] | [ ] |
| Achievement of immune reconstitution | [ ] | [ ] |
| Reduction in HIV transmission | [ ] | [ ] |
| Reduction in viral load | [ ] | [ ] |
| 1. **What is/are the criteria for initiating HAART?** |  |  |
| CD4 level 500 or below | [ ] | [ ] |
| WHO Stage 3 and 4 irrespective of CD4 count | [ ] | [ ] |
| Co-existing TB Infection | [ ] | [ ] |
| 1. **I understand the WHO clinical staging of HIV/AIDS** | [ ] | [ ] |
| 1. **The minimum number of ARVs that should be included in an ideal HIV/AIDS regimen is two.** | [ ] | [ ] |
| 1. **What is/are the approved combination(s) of ARVs to be used in HAART regimen?** |  |  |
| Efavirenz + Lamivudine + Tenofovir | [ ] | [ ] |
| Abacavir + Lamivudine + Efavirenz | [ ] | [ ] |
| Lopinavir/ritonavir + Lamuvidine +Zidovudine | [ ] | [ ] |
| Zidovudine+Lamuvudine+Emtricitabine | [ ] | [ ] |
| 1. **Drug that is added as booster to Lopinavir is Zidovudine** | [ ] | [ ] |
| 1. **The ideal storage condition for ARVs is 2 - 8^0^c** | [ ] | [ ] |

**Section 3: Attitudes and Present Involvement**

| **Involvement: Indicate whether you carry out each of the activities below in your premise;** | **Yes** | **No** |
| --- | --- | --- |
| 1. Encounter with HIV clients | [ ] | [ ] |
| 1. Advice HIV clients on their medications | [ ] | [ ] |
| 1. Encounter with suspected cases of HIV infection | [ ] | [ ] |
| 1. Stocking of HIV test kit | [ ] | [ ] |
| 1. Stocking of ARVs | [ ] | [ ] |
| 1. Stocking of condoms | [ ] | [ ] |

| 1. **What do you do when you encounter suspected HIV/AIDS in your premise?** | **Yes** | **No** |
| --- | --- | --- |
| Educate, counsel and reassure the patient | [ ] | [ ] |
| Recommend drugs for other associated minor conditions and then, document | [ ] | [ ] |
| Refer the patient to the hospital | [ ] | [ ] |

| **Rate your extent of agreement/willingness in the following statements;** | **Strongly disagree/Not**  **willing** | **Disagree/**  **Not**  **willing** | **Undecided** | **Agree/**  **willing** | **Strongly agree/**  **willing** |
| --- | --- | --- | --- | --- | --- |
| 1. Community pharmacist have a role to play in provision of HIV/AIDS Services | [ ] | [ ] | [ ] | [ ] | [ ] |
| 1. Your willingness to use your premise as a registered service delivery site for HIV/AIDS patients? | [ ] | [ ] | [ ] | [ ] | [ ] |
| 1. i. Training and sensitization lectures to community pharmacists are necessary to enhance involvement in HIV/AIDS care | [ ] | [ ] | [ ] | [ ] | [ ] |
| ii. Payment/remuneration for HIV/AIDS care services will motivate the pharmacists in this service | [ ] | [ ] | [ ] | [ ] | [ ] |
| iii. Government supports and other incentives are necessary for the smooth delivery of HIV/AIDS care services | [ ] | [ ] | [ ] | [ ] | [ ] |

**Section 4: Skills and Readiness Assessment**

| **Readiness** | **No** | **Yes** |
| --- | --- | --- |
| 1. Does your pharmacy have a functional backup generator to support the electricity whenever there is power outage? | [ ] | [ ] |
| 1. Does your pharmacy premise have counseling section where you interact with clients privately? | [ ] | [ ] |
| 1. Does your counseling section have auditory and visual privacy | [ ] | [ ] |
| 1. Do you use patient information sheet/Book for collection of information from patients in your pharmacy | [ ] | [ ] |
| **Perceived Skill: do you have the adequate skill to carry out the following activities?** | **No** | **Yes** |
| 1. Educating target population on HIV | [ ] | [ ] |
| 1. Demonstrable skill on how to carry out HIV rapid test | [ ] | [ ] |
| 1. Counseling on importance of adherence to HIV medication | [ ] | [ ] |
| 1. Referral of HIV client for care | [ ] | [ ] |
| 1. HIV care, documentation and reporting | [ ] | [ ] |
